# Hydrogen bond networks determine emergent mechanical and thermodynamic properties across a protein family

**DOI:** 10.1186/1752-153X-2-17

**Published:** 2008-08-12

**Authors:** Dennis R Livesay, Dang H Huynh, Sargis Dallakyan, Donald J Jacobs

**Affiliations:** 1 Department of Computer Science and Bioinformatics Research Center, University of North Carolina at Charlotte, Charlotte, NC, USA; 2 Physics and Astronomy Department, California State University at Northridge, Northridge, CA, USA; 3 Department of Physics and Optical Science, University of North Carolina at Charlotte, Charlotte, NC, USA

## Abstract

**Background:**

Gram-negative bacteria use periplasmic-binding proteins (bPBP) to transport nutrients through the periplasm. Despite immense diversity within the recognized substrates, all members of the family share a common fold that includes two domains that are separated by a conserved hinge. The hinge allows the protein to cycle between open (apo) and closed (ligated) conformations. Conformational changes within the proteins depend on a complex interplay of mechanical and thermodynamic response, which is manifested as an increase in thermal stability and decrease of flexibility upon ligand binding.

**Results:**

We use a distance constraint model (DCM) to quantify the give and take between thermodynamic stability and mechanical flexibility across the bPBP family. Quantitative stability/flexibility relationships (QSFR) are readily evaluated because the DCM links mechanical and thermodynamic properties. We have previously demonstrated that QSFR is moderately conserved across a mesophilic/thermophilic RNase H pair, whereas the observed variance indicated that different enthalpy-entropy mechanisms allow similar mechanical response at their respective melting temperatures. Our predictions of heat capacity and free energy show marked diversity across the bPBP family. While backbone flexibility metrics are mostly conserved, cooperativity correlation (long-range couplings) also demonstrate considerable amount of variation. Upon ligand removal, heat capacity, melting point, and mechanical rigidity are, as expected, lowered. Nevertheless, significant differences are found in molecular cooperativity correlations that can be explained by the detailed nature of the hydrogen bond network.

**Conclusion:**

Non-trivial mechanical and thermodynamic variation across the family is explained by differences within the underlying H-bond networks. The mechanism is simple; variation within the H-bond networks result in altered mechanical linkage properties that directly affect intrinsic flexibility. Moreover, varying numbers of H-bonds and their strengths control the likelihood for energetic fluctuations as H-bonds break and reform, thus directly affecting thermodynamic properties. Consequently, these results demonstrate how unexpected large differences, especially within cooperativity correlation, emerge from subtle differences within the underlying H-bond network. This inference is consistent with well-known results that show allosteric response within a family generally varies significantly. Identifying the hydrogen bond network as a critical determining factor for these large variances may lead to new methods that can predict such effects.

## Background

The ATP-Binding Cassette (ABC) transporting system is essential for uptake of specific nutrients by bacteria [1]. ABC transporters are usually made up of a soluble-binding protein, a transmembrane protein, and an ATP-binding protein. The soluble-binding protein, also known as bacterial periplasmic-binding protein (bPBP), resides in the periplasm of Gram-negative bacteria. It selectively binds small molecules (i.e., amino acids, inorganic phosphate, or sugars), and carries them to the transmembrane component. As the loaded bPBP docks to the transmembrane unit, its conformation changes such that the substrate can be transferred to the transmembrane protein, and the bPBP is functionally recycled [2].

A large number of resolved x-ray crystal structures indicate that bPBP is composed of two domains connected by a flexible polypeptide linker that allows the protein to cycle between open and closed conformations. At equilibrium, the apo structure fluctuates between both conformations. Introduction of ligand stabilizes the closed conformation and thus concomitantly shifts the equilibrium. The ubiquitous bPBP hinge-bending motion makes them attractive targets for many practical applications, including drug delivery [3] and biosensors [4]. They are attractive drug delivery agents because the equilibrium between open and closed is sensitive to local environment (i.e., pH, temperature, or presence of interacting enzymes). Thus, it is feasible to engineer bPBP to bind to a drug of interest and then release it when the complex reaches a desired cell or compartment where conventional drug delivery methods fail to reach [3]. The ligand-mediated hinge-bending motion in bPBPs can also be used to design fluorescent biosensors by covalently attaching fluorphore molecules so that fluorescence intensity and/or wavelength changes as a result of hinge-bending motion [4]. In this way, the high specificity of binding and ligand-mediated conformational changes in bPBPs can be exploited to monitor for the presence of a specific ligand.

A complete understanding of bPBP requires both mechanical and thermodynamic descriptions. Mechanical descriptions are necessary in order to detail changes in flexibility and mechanical couplings upon ligand binding, whereas thermodynamic descriptions are necessary to account for an ensemble of conformations associated with the apo and ligated states. Most computational methods focus only on one of the two phenomena. There are several common methods to explore protein thermodynamics (i.e., Monte Carlo sampling [5], a variety of Ising-like models [6,7] such as COREX [8], and free energy decomposition schemes [9-11]). Unfortunately, these methods generally fail to reproduce experimental thermodynamic response (i.e., excess heat capacity profiles). In addition, while most of these methods do consider an ensemble of conformations, they lack descriptions of mechanical couplings between sites within the protein. On the other hand, mechanical models (i.e., FIRST [12] and Elastic Network Models (ENMs) [13]) do provide detailed flexibility information, yet they completely lack any thermodynamic considerations. In principle, all-atom molecular dynamics (MD) simulations could be used to predict any mechanical and thermodynamic property of a protein. However, in practice, MD is much too computationally expensive to explore the thermodynamic limit [14]. Consequently, Go-like models have been the mainstay approach that employs molecular dynamics simulation in conjunction with simplified molecular mechanics potentials tuned to the native state (or multiple states) [15,16].

In this report, we employ a minimal Distance Constraint Model (mDCM) to investigate stability/flexibility relationships of four homologous bPBPs. We choose to use the mDCM among the available methods because; (*i*.) it uniquely synthesizes mechanical and thermodynamic descriptions, (*ii*.) its predictions compared to experiment have consistently achieved overall good agreement across a diverse population of proteins [18,15,17], and (*iii*.) it is a tractable modeling paradigm requiring just ~10 minutes of compute time for a 300 residue protein per thermodynamic condition.

## Experimental

### The distance constraint model

The DCM is based on a free energy decomposition scheme combined with constraint theory, such that microscopic interactions are represented as mechanical distance constraints. Each distance constraint is associated with an enthalpic and entropic contribution [17]. A Gibbs ensemble of accessible microstates is defined by a set of topologically distinct mechanical frameworks. A single mechanical framework encompasses an ensemble of all accessible molecular conformations that are consistent with a specified set of distance constraints. As a result, each framework is defined by the topology of constraint placement, and the enthalpy of the framework is calculated as a linear sum of enthalpy components over all constraints present. While preserving a specified distance constraint topology, the protein will sweep over its accessible phase space as it changes shape (geometry). As the model is quite simple, it is assumed that as long as the constraint topology does not change, different geometries consistent with the specified constraints are degenerate in enthalpy. As a result, the conformational entropy of each mechanical framework is meaningful. It has been a major problem that conformational entropy is generally a nonadditive property of free energy decompositions [18]. As explained below, we account for this nonadditive property using network rigidity to obtain a good upper bound estimate of conformational entropy by summing entropy components over a preferential set of independent constraints.

Specifically, the DCM decomposes the Gibbs free energy of a single framework into sums of different types of microscopic interactions also modeled as one (or more) distance constraint(s). Each interaction type, *t*, is assigned enthalpic and entropic values (*H*_*t *_and *S*_*t*_, respectively). Enthalpic contributions are linearly summed, whereas entropic contributions are only summed over independent constraints, which are identified by a network rigidity algorithm. Key to this rigidity algorithm is to recursively build up a mechanical framework one constraint at a time and, during this process, to identify which constraints are independent. Interestingly, the set of independent constraints that this recursive process identifies is not unique, because the identification of redundancy depends on the specific ordering of constraint placement. Recalling that only independent constraints will contribute to the total conformational entropy, it is clear that any order provides an upper bound estimate to the true conformational entropy. Due to the recursive nature of the rigidity algorithm, a rigorous lowest upper bound estimate is achieved by placing the lowest entropy constraints preferentially before any other constraints with higher entropy values.

Regarding the long-range nature of network rigidity as an underlying mechanical interaction captures the nonadditive nature of conformational entropy. The free energy for a given framework, *F*, is calculated by:

(1) $$G(F)=\sum_{t}H_{t}N_{t}-T\sum_{t}S_{t}I_{t}$$

where *N*_*t *_is the total number of interactions of type *t*, and *I*_*t *_is the total number of *independent constraints *of type *t*, which is uniquely identified because of the preferential ordering. Subsequently, the partition function can be constructed from all possible frameworks. Detailed descriptions of how to calculate the Gibbs free energy, free energy landscapes, and partition functions using the DCM can be found in several publications [19-22]. Solving the DCM can be done using a number of different techniques. For example, transfer matrix methods have been applied to the helix to coil transition [23,24]. Additionally, we recently developed an *ab initio *method based on analytical solutions of the configuration integral that model distance constraints as delta functions (unpublished). Here we employ a novel non-homogenous mean field approximation that we previously developed [19,25].

### Free energy decomposition

Within the mDCM, the microscopic interactions explicitly modeled are covalent bonds, hydrogen bonds (H-bonds), and torsional-forces; note that salt bridges represent a special case of H-bonds. The covalent bonds within a protein do not break/form due to thermal fluctuations, and are therefore quenched constraints (ever present). Quenched constraints require no parameterization for their enthalpy and entropy contributions because they only shift the free energy of a protein by a constant. The mDCM is based on three types of fluctuating interactions that need parameterization (H-bonds and two types of torsion-forces). An enthalpic and entropic value is associated with each interaction (see Table 1). Intramolecular H-bond enthalpies are described using a common multi-atom empirical potential [26] that depends on quantum mechanical hybridization, and on the local environment defined by all atom positions. The H-bond potential energy function ensures that the enthalpy of an intramolecular H-bond ranges from -8 to 0 kcal/mol.

**Table 1:** Parameterization of the mDCM.^1^

Parameter	Value^2^	Treatment	Description
*U_*hb*_*	Context dependent	Empirical potential [26]	Intramolecular H-bond energy
*γ*_*max*_	1.99	Constant	Linearly relates H-bond pure entropy to its energy
*u*	-1.91	Fitting	H-bond to solvent energy upon breaking of intramolecular H-bond
*v*	-0.64	Fitting	Native torsional constraint energy
*δ_*nat*_*	1.42	Fitting	Native torsional constraint pure entropy
*δ_*dis*_*	2.56	Constant	Disordered torsional constraint pure entropy

^1^Note that the disordered torsional constraint energy is the reference energy and is equal to 0.00. The mDCM ignores potential entropic contributions on the formation of H-bonds to solvent. ^2 ^The values of the three fitting parameters were determined by fitting HBP to experimental *C*_*p *_curves [27].

Different interaction types are modeled by a different number of distance constraints. The two torsion-force interactions are each modeled as one distance constraint, while the multi-particle H-bond and covalent bond interactions are each modeled as five constraints [12]. The entropic cost, *γ*_*env*_, for each independent constraint representing the H-bond is modeled as a linear relationship to the H-bond enthalpy by *γ*_*env *_= *γ*_*max *_(1+E/8), where *γ*_*max *_is a fitting parameter. It follows that 0 ≤ *γ*_*env *_≤ *γ*_*max *_is the allowed entropy range per H-bond distance constraint. Each of the five distance constraints modeling a single H-bond are assigned the same entropy value. Sigma covalent bonds are free to rotate and are partitioned into native-like or disordered conformational states that represent an Ising-like (binary) discrimination of torsion-forces. The (native, disordered) conformation includes all dihedral angles that are (similar to, substantially differing from) those defined in the native three-dimensional structure. Table 1 lists enthalpies and entropies for the torsion-force constraints.

### Free energy landscape

**Figure 1:**
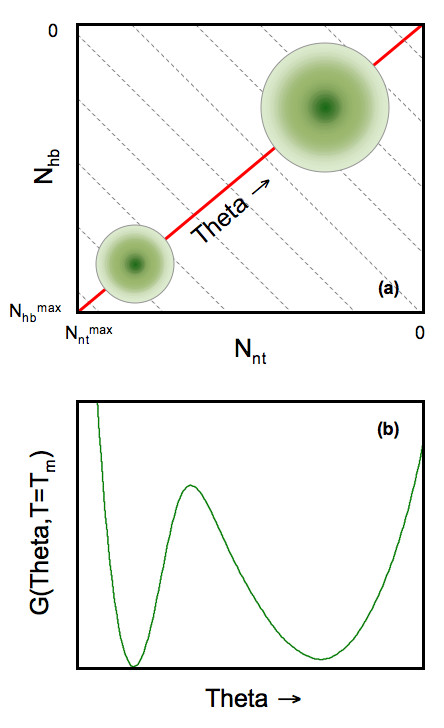
**(a) **Cartoon of the free energy landscape in two-dimensional constraint space. Each point on the two-dimensional grid defines a macrostate, *(N_*nt*_, N_*hb*_)*, where the free energy, *G(N_*nt*_, N_*hb*_)*, is calculated. The green shading is meant to describe the native (lower-right) and unfolded (upper-left) basins within the free energy landscape. (Notice that the axes are decreasing from bottom to top and left to right.) At times it is convenient to express the free energy as a function of a one-dimensional flexibility order parameter, *θ(N_*nt*_, N_*hb*_)*. Grey dashed lines represent (approximate) fronts of constant global flexibility due to tradeoff between two constraints types. The red line denotes the shortest path crossing a single saddle from the unfolded to folded basins. **(b) **An example one-dimensional free energy landscape highlights the straddling barrier that must be crossed as the protein transitions between folded and unfolded.

The free energy landscape is defined using two order parameters: the number of native torsion-force constraints, *N*_*nt*_, and the number of H-bond constraints, *N*_*hb *_(see Fig. 1a). The free energy of each macrostate, *G(N*_*nt*_, *N*_*hb*_*)*, is calculated using a mean-field approximation [19] by Monte Carlo sampling over frameworks satisfying the two order parameters; as few as 200 samples are needed for good statistics. Using the enthalpic and entropic parameters in Table 1, the free energy of a given macrostate is calculated by:

(2) *G*(*N*_*nt*_, *N*_*hb*_) = *U*_*hb*_(*N*_*hb*_) - *uN*_*hb *_+ *vN*_*nt *_- *T *[*S*_*c*_(*N*_*nt*_, *N*_*hb*_) + *S*_*m*_(*N*_*nt*_, *N*_*hb*_)]

where *U*_*hb *_is the intramolecular H-bond energy, *u *is an average H-bond energy to solvent, *v *is the energy of a native-like torsion angle, *S*_*c*_*(N*_*nt*_, *N*_*hb*_*) *is the conformational entropy, and *S*_*m*_*(N*_*nt*_, *N*_*hb*_*) *is the mixing entropy of the macrostate associated with the number of ways of distributing *N*_*nt *_native-torsions and *N*_*hb *_H-bonds within the protein. While not specified here, *S*_*c*_*(N*_*nt*_, *N*_*hb*_*) *is a linear function of *γ*_*env*_, *δ*_*nat*_, *δ*_*dis*_, and *I*_*t*_, where *t *denotes each of the three fluctuating constraint types (see Table 1) [19]. In most systems investigated previously [19-22], hysteresis is observed near the melting temperature, *T*_*m*_, meaning there are two minima within the free energy landscape corresponding to the native and unfolded ensembles. These two free energy basins are schematically shown in Fig. 1a and will appear when the protein exhibits two-state folding characteristics.

Values of the three free parameters {*u*, *v*, *δ*_*nat*_} are determined by fitting to experimental heat capacity (*C*_*p*_) curves from differential scanning calorimetry (DSC) [21]. To the best of our knowledge, the DCM is the only microscopic computational modeling scheme that can quantitatively reproduce experimental protein heat capacity curves. *C*_*p *_is directly computed from the enthalpic fluctuations, ⟨Δ*H*^2^⟩, across the ensemble using the following relationship:

(3) $$C_{p}=\frac{dH}{dT}=\frac{\langle \Delta H^{2}\rangle }{RT^{2}}$$

The fluctuations are computed from the enthalpic portions of Eq. (2) using:

(4) $$\langle \Delta H^{2}\rangle =\sum_{N_{nt},N_{hb}}{\left(H-\langle H\rangle \right)}_{N_{nt},N_{hb}}^{2}\;e^{-\frac{G(N_{nt},N_{hb})}{RT}}$$

Simulated annealing is used to search through the parameter-space to find the best-fit solution(s) to the experimental *C*_*p *_data. In previous works [19,21], two of the parameters (*γ*_*max *_and *δ*_*dis*_) listed in Table 1 have been fixed, and are now treated as transferable. For a given thermodynamic condition, the entire free energy landscape and many thermodynamic response functions are calculated in a matter of ~10 minutes for a 300-residue protein on a desktop computer using one CPU. A workflow diagram for the mDCM is provided in Additional file 1.

### Ensemble averaging network rigidity

In addition to the thermodynamic descriptions, the DCM also computes mechanical properties of a protein. The mechanical and thermodynamic aspects to the problem are actually intertwined such that one cannot be calculated without the other. In virtually instantaneous compute times, graph rigidity algorithms identify rigid and flexible regions, independent constraints, redundant constraints, and correlated motions. FIRST is capable of doing these calculations in a fraction of a second for a 300-residue protein. In contrast, the DCM runs in matter of minutes, because these rigidity calculations are repeated millions of times using Monte Carlo sampling throughout the various nodes in the free energy landscape. A large number of quantities that describe the mechanical nature of the protein can be derived (see below), and these properties are averaged over many accessible frameworks. Randomly perturbing the constraint topology initially defined by the native template structure generates the ensemble of these accessible frameworks.

The rigidity information from distinct constraint topologies is appropriately averaged using Boltzmann weights, given as exp(-*G*(*N*_*nt*_, *N*_*hb*_), within the free energy landscape node of interest. It is worth noting that the program FIRST [12], a popular implementation of network rigidity for proteins, uses an *ad hoc *sliding energetic cut-off threshold, *E*_*c*_, to determine when H-bond constraints are present or not. FIRST is an athermal model where each constraint is either present or not; meaning, it does not allow for fluctuations. When a constraint is present, regardless of what it is being used to model (i.e., covalent vs. noncovalent bond), it is treated as equivalent. In the DCM, large numbers of accessible constraint topologies are sampled, their associated free energies calculated, and then the native free energy basin is identified. As such, the most probable states (free energy basins) are determined, which eliminates the arbitrariness of *E*_*c*_.

Once mDCM parameterization is achieved, a complete set of mechanical and thermodynamic quantities can be calculated. A large number of mechanical quantities (including: rigid cluster decomposition, independent and redundant constraints, rigid cluster susceptibility, global flexibility, local flexibility index, and flexibility propagation) are evaluated in a thermodynamically meaningful way. Moreover, averages of local mechanical properties can be used to identify long-range couplings between sites (i.e., allosteric communication). As such, the DCM provides a natural mechanism to evaluate Quantitative Stability/Flexibility Relationships (QSFR) [22].

### Selection of DCM parameters

Remarkably, backbone flexibility predicted by FIRST with suitable *E*_*c*_, applied to a single constraint topology (i.e. derived from the native state template structure), is usually similar to the result from the DCM [21]. This is an indication that there is a most probable state that is sharply peaked around typical backbone flexibility characteristics. Ensemble averaging makes rigidity/flexibility predictions robust, while eliminating the arbitrariness in *E*_*c*_. Nevertheless, the DCM has three free parameters {*u*, *v*, *δ*_*nat*_} that are obtained by fitting to heat capacity, for example. From previous [22] and unpublished work, we find that rigidity/flexibility properties are largely insensitive to a ± 15% deviation in these three free parameters, and there is a corresponding variance of about ± 10% in the predicted *T*_*m *_(corresponding to ± 35 K). Given these sensitivity levels, the fitting parameters determined from one reference protein structure (based on its measured *C*_*p*_) is then applied on all similar proteins, such as those from the same family. In this work, the three fitting parameters {*u*, *v*, *δ*_*nat*_} are taken from previous work [19], where they were determined by fitting Histidine Binding Protein (HBP) to the *C*_*p *_data as reported in reference [27]. As such, we are able to characterize emergent mechanical and thermodynamic properties across a series of proteins in a consistent way. Similarities and differences in both mechanical and thermodynamic properties will therefore reflect structural differences found in these proteins.

### Relating thermodynamic and mechanical quantities

As described above, an ensemble of mechanical frameworks is needed to obtain thermodynamic information from a statistical mechanics description of the system, and mechanical quantities are averaged over this ensemble. Moreover, correlation functions describing detailed rigidity/flexibility properties within a protein can be calculated based on probability measures over the ensemble of constraint topologies. We generically refer to all thermodynamic and mechanical information taken together as QSFR. The QSFR measures employed in this work are now described.

The free energy landscape is described, as defined in Eq. 2, by the function *G(N*_*nt*_, *N*_*hb*_*)*. The macrostate of the protein is specified by *(N*_*nt*_, *N*_*hb*_*)*, which defines a node within Fig. 1a. Besides free energy, other physical properties are expressed as a function of the macrostate. For each node, consider a physical property given by *x(N*_*nt*_, *N*_*hb*_*)*, which is already an average over the sub-ensemble of all constraint topologies associated with the macrostate. One such property of particular importance is the global flexibility of a protein, which is the number of independent degrees of freedom per residue. We call this quantity the global flexibility order parameter, defined by *θ*' (*N*_*nt*_, *N*_*hb*_) ≡ ⟨*I*_*dis *_(*N*_*nt*_, *N*_*hb*_)⟩/*n*, where *n *is the number of residues in the protein. The quantity ⟨*I*_*dis*_(*N*_*nt*_, *N*_*hb*_)⟩ is the average number of independent disordered torsion constraints over a sub-ensemble of constraint topologies of the macrostate.

Collapsing the free energy landscape in constraint space onto the global flexibility order parameter makes a direct connection between free energy of a protein and its flexibility. The process entails calculating a partition function, *Z(θ)*, given by:

(5) $$Z(\theta )=\sum_{N_{nt},N_{hb}}B\left(\theta ,N_{nt},N_{hb}\right)\;e^{-\frac{G(N_{nt},N_{hb})}{RT}}$$

where *B(θ, N*_*nt*_, *N*_*hb*_*) *is a binning function. The binning function is 1 whenever *-Δθ < |θ-θ '(N*_*nt*_, *N*_*hb*_*)| *≤ *Δθ*, and 0 otherwise. Note that *Δθ *is a tolerance used for bin size, which has been set to 0.005 because we plot functions of *θ *in increments of 0.01. Thus, all nodes of the original two-dimensional landscape must fall into one, and only one, *θ *bin. The free energy of the protein as a function of its global flexibility is given as *G*(*θ*, *T*) = -*RT*ln*Z*(*θ*). Consequently, this mapping straightforwardly results in a simple one dimensional free energy landscape.

At the *T*_*m*_, proteins that fold via two-state kinetics will have a free energy landscape that looks like a "W". That is, *G(θ, T*_*m*_*) *will have two minimum and an intervening barrier that separates the two minima. In general, it is difficult to interpret an order parameter as a reaction coordinate. We have suggested that the *θ *may be a good reaction coordinate, but have not studied kinetics to confirm this. However, it is instructive to explain features about *θ *that would be required if it did indeed serve as a reaction coordinate. Within the mDCM, there are two types of fluctuating constraints, which define the two-dimensional free energy landscape shown in Fig. 1a. Proceeding from left to right, *θ *will decrease since the protein rigidifies as more native torsion force constraints are added. Similarly, progressing from bottom to top, *θ *will decrease in the same manner as more H-bonds are added. Consequently, the locus of points along any one of the light grey lines will have nearly constant *θ *because a reduction of rigidity in one direction is offset by an increase in the other. The lower left-hand side of the plot coincides to small values of *θ*, whereas the upper-right coincides with larger values. The red line demonstrates the most direct route between the two free energy basins, over a single saddle. The saddle (Fig. 1b) reflects a state with a mixture of rigid and flexible regions coexisting, which involved a nucleation process of either forming rigid regions upon folding, or breaking apart rigid regions upon unfolding.

From simple geometric considerations, and the construct of the mDCM, the path along the global flexibility order parameter must always cross the saddle. This motivates calculating other physical quantities in terms of *θ*. We calculate the average properties of a physical property, *x(N*_*nt*_, *N*_*hb*_*)*, over a restricted ensemble based on the protein's global flexibility, given by:

(6) $$X(\theta ,T)=\sum_{N_{nt},N_{hb}}x\left(N_{nt},N_{hb}\right)\;\frac{B(\theta ,N_{nt},N_{hb})\cdot e^{-\frac{G(N_{nt},N_{hb})}{RT}}}{Z(\theta )}$$

For example, it is of interest to select *θ *to be one of the following: *θ*_*nat*_, *θ*_*TS*_, or *θ*_*unf*_, which identifies the native basin, the assumed transition state maximum, and the unfolded minimum, respectively, in the function *G(θ,T*_*m*_*)*. Selecting *θ*_*nat *_allows us to compare mechanical properties that are averaged over a native state ensemble. Note, in the results presented herein, the native state ensemble is extended slightly to include a range over *θ *near *θ*_*nat *_(equation not shown), which increases statistics and accounts for all variance within the entire free energy basin. Nevertheless, expanding the range of *θ *does not lead to any qualitative differences within QSFR.

The value of *θ*_*nat *_gives the average number of independent degrees of freedom per residue that is available to the protein in its native state. We also probe where these degrees of freedom are localized, and quantify the flexible and rigid parts of the protein. To do so, we define a flexibility index [12,19,21] to describe the extent of flexibility/rigidity along the protein backbone. The flexibility index of residue *i*, *f*_*i*_, is defined by Eq. 6 with X → *f*_*i *_and $x\to (\rho_{i}^{idf}-\rho_{i}^{rdc})$. The quantities $\rho_{i}^{idf}$ and $\rho_{i}^{rdc}$ are functions of the macrostate, $\rho_{i}^{idf}$*(N*_*nt*_, *N*_*hb*_*) *and $\rho_{i}^{rdc}$*(N*_*nt*_, *N*_*hb*_*)*, where they are the average densities of independent degrees of freedom and redundant constraints, respectively, at residue *i*. These quantities have the same definition as used in FIRST [12], except here they are averaged uniformly over a sub-ensemble of constraint topologies that have *N*_*nt *_native torsion constraints, and *N*_*hb *_intramolecular H-bonds in the network. After the second averaging procedure, we obtain the flexibility index for the native sate of the protein as a function of temperature. The range of *f*_*i*_*(T) *over all residues in a protein is always less than 1, and almost always greater than -1. A negative flexibility index indicates that the region is over-constrained, meaning it is rigid with more constraints than necessary to be rigid. A more negative value implies a greater density of redundant constraints. A positive flexibility index indicates that the region is flexible, meaning it is capable of motion involving a certain number of degrees of freedom. A more positive value implies a greater density of degrees of freedom to describe the motion. A flexibility index near 0 indicates a marginally rigid region having neither excess constraints nor degrees of freedom. However, due to the ensemble averaging, these marginal regions are often split between being slightly over-constrained and slightly flexible. Marginal regions are particularly interesting, as they have a high degree of susceptibility to perturbations in the network.

While *f*_*i *_is a measure of flexibility along the backbone, cooperativity correlations are defined to describe intramolecular couplings between sites within the protein. These couplings are both thermodynamic and mechanical in nature. Cooperativity correlation between two residues *i *and *j*, *C*_*ij*_, describes both rigid and flexible couplings. Flexibility correlation quantifies the extent that two residues, across the thermodynamic ensemble, are simultaneously flexible and connected by a path in which flexibility can propagate. Conversely, rigidity correlation quantifies the extent that two residues are simultaneously within the same rigid cluster. Zero cooperatively correlation indicates that two sites are neither flexibly nor rigidly coupled. Frequently, the most flexible portions of a protein (e.g., the N- or C-termini) have no correlation to the rest of the protein due to the fact that the mechanical aspect of the definition (meaning that two sites must be connected by a path in which flexibility can propagate) is not satisfied. The correlation matrices are functions of temperature and global flexibility too. Since mechanical response will be temperature dependent, we need to address at what temperature should be used when comparing mechanical response of different proteins, but are part of the same family. In general, we work at *T*_*m *_because, first, there is weak temperature dependence in any of the native ensemble averaged mechanical properties. Second, and more importantly, the *T*_*m *_of each considered protein establishes a corresponding location along the folding transition, which is in contrast to a set absolute temperature, of say 320 K. Due to weak temperature dependence, the exact value of *T*_*m *_is not that important as far as the mechanical properties are of concern. Therefore, the error in predicting *T*_*m *_is not of much concern for making QSFR comparisons across similar proteins.

### Protein structure preparation

The employed H-bond potential [26] requires explicit hydrogen atoms. Most methods of adding hydrogen atoms assume pH = 7 and do not account for changes in residue-specific titration profiles based on electrostatic interactions or solvent conditions. Here, the pH of the *C*_*p *_data used to parameterize the mDCM is 8.3. Consequently, we use the *pK*_*a *_calculation implemented within the University of Houston Brownian Dynamics suite of programs [28] to calculate whether or not a titratable residue should be protonated or not at a specific pH (see [29,30] for details of the calculation). Hydrogen atoms are (kept, removed) if their probability for protonation is (greater, lesser) than 50 percent.

## Results

### The bacterial periplasmic binding protein family

**Figure 2:**
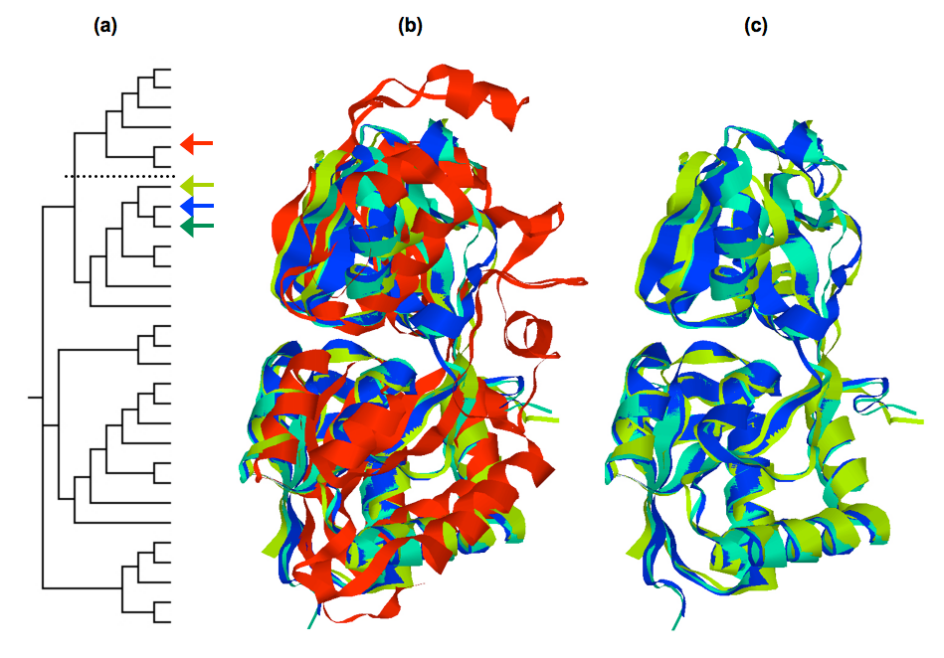
**(a) **Phylogenetic tree of representatives from the 29 protein classes within the bPBP family. The four proteins investigated herein are highlighted (Blue = LAOBP, Green = HBP, Purple = GBP, and Red = PhBP). **(b) **Structure superposition of the four binding proteins (color-coding the same). PhBP has been removed in **(c) **to highlight the conservation within the three amino acid binding proteins.

Within the Structural Classification of Proteins (SCOP) database [31], there are 29 different binding protein classes within the bPBP family, which SCOP calls the phosphate binding protein-like family. Several classes within the family include multiple species orthologs, and within each ortholog, many binding proteins have been crystallized within different states (i.e., presence/absence of ligand, wild-type vs. mutant, etc.). Fig. 2a shows the neighbor-joining phylogenetic tree built from the Probalign [32,33] multiple sequence alignment of 29 representative structures. Ideally, we would like to perform a comparative QSFR analysis across all 29 binding protein classes; however, pragmatic considerations make this impractical for manual manipulations. Since a large scale comparison will need automation based on prior experience, we extend our previous comparative QSFR analysis of a mesophilic/thermophilic RNase H pair [22] to four different binding proteins. There is experimental *C*_*p *_data for only one member of the family, the histidine binding protein (HBP). Three of the homologs, which include lysine/arginine/ornithine binding protein (LAOBP) [34], glutamine binding protein (GBP) [35] and HBP [36], represent a closely related subfamily of amino acid binding proteins. Juxtaposed to this close-knit group is the distantly related phosphate binding protein (PhBP) [37], the namesake of the SCOP family, which provides a point of reference over larger evolutionary distances. In order to circumvent parameterization issues, we apply the HBP parameterization to all four homologs, which we have previously demonstrated to be a satisfactory option [22], especially when considering mechanical response. Two of the binding proteins have been crystallized in both the presence and absence of ligand, whereas the other two have only been crystallized in the presence of ligand. Table 2 lists the PDB ID's and other relevant information of the six structures investigated herein.

**Table 2:** Descriptions of the structures used in this report.^1^

	LAOBP		HBP	GBP		PhBP
Source	*S. typhimurium*		*E. coli*	*E. coli*		*E. coli*

PDB ID	1LST	2LAO*	1HSL	1WDN	1GGG*	1IXH

Bound ligand^2^	Lysine	n/a	histidine	glutamine	n/a	phosphate
Number of residues	238	238	238	223	220	321
Large domain^3^	1–90, 191–240		1–90,191–238	4–88, 182–226		1–75,255–321
Small domain	91–190		91–190	89–181		76–254
Resolution (Å)	1.80	1.90	1.89	1.94	2.30	0.98
Number of H-bonds	352	357	327	327	293	504
Number of H-bonds to substrate	5	n/a	6	9	n/a	7
Total H-bond energy (Kcal/mol)^4^	-942.11	-875.20	-714.00	-757.28	-633.40	-1274.28
Avg. H-bond energy (kcal/mol)	-2.68	-2.45	-2.18	-2.32	-2.16	-2.53
Std. dev. H-bond energy (kcal/mol)	2.34	2.27	2.12	2.22	2.05	2.30

^1^All four bPBP homologs have been crystallized in the ligated conformation. Additionally, two bPBP homologs (marked with asterisks) have been crystallized in the open apo conformation. ^2 ^Non-biologically relevant co-crystallized solutes (i.e., metal ions) are ignored. ^3 ^Domain boundaries are identified from the ligated structures using PDP [40]. ^4 ^Total H-bond energy is simply the sum of the energies for each H-bond observed within the structure.

As expected from the phylogeny, structural superposition of all four ligated binding proteins (see Fig. 2b) confirms that the three amino acid binding proteins are much more structurally similar to each other than to PhBP. In Fig. 2c, PhBP is removed to highlight the similarity within the amino acid binding proteins. Table 3 provides a more quantifiable description of (dis)similarity across the four binding proteins. Therein, pairwise root mean square deviation (RMSD) are computed using the combinatorial extension algorithm [38], and percent sequence identities are computed from the pairwise global alignment using a dynamic programming algorithm [39]. Secondary structure topologies are mostly conserved across the four binding proteins considered here. However, across the entire bPBP family, several members have noncanonical secondary structure topologies, which indicates that a structure alignment of the complete family would be even less conserved.

**Table 3:** All to all pairwise comparisons of the four ligated bPBP structures.^1^

	LAOBP	HBP	GBP	PhBP
LAOBP	---	1.1 Å	1.5 Å	4.2 Å
HBP	69.9%	---	1.9 Å	4.4 Å
GBP	30.3%	29.3%	---	4.1 Å
PhBP	16.6%	17.5%	17.1%	---

^1^Numbers above the diagonal represent pairwise RMSD (Å) between each structure (calculated using the combinatorial extension algorithm [38]), whereas numbers below the diagonal represent percent residue identity, as calculated from each global pairwise alignment.

**Figure 3:**
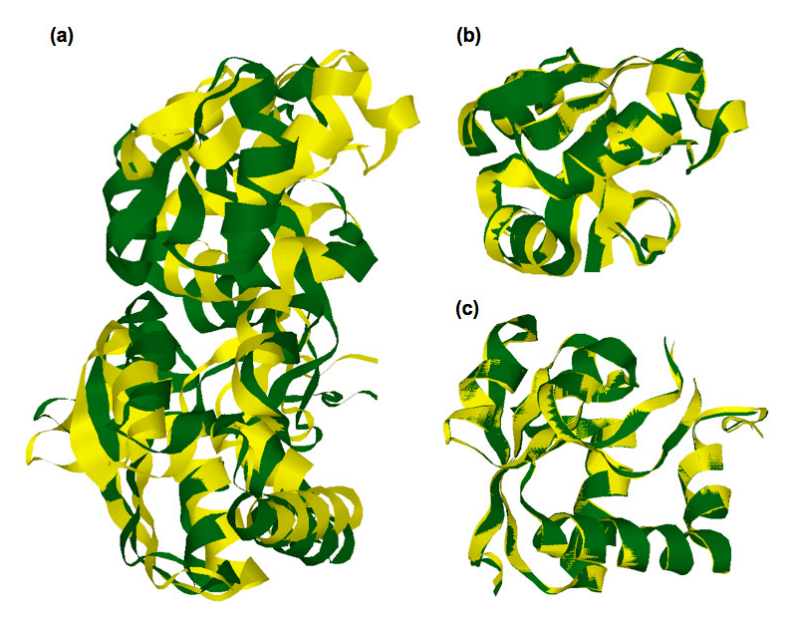
**(a) **Structure superposition of the ligated (blue) and apo (red) LAOBP conformations as calculated by the combinatorial extension algorithm. The overall RMSD between the two conformations is 3.5 Å. Structure superposition of the **(b) **large and **(c) **small domains is also provided. The domain specific RMSDs are 0.6 Å and 0.4 Å, respectively.

From the superposition of the apo and ligated LAOBP conformations (Fig. 3a), it is clear that there is pronounced conformational changes on ligand binding. In fact, the RMSD between the two LAOBP conformations is 3.5 Å. Similarly, the pairwise RMSD between the GBP pair is 4.0 Å. Although the RMSD values reported in Table 3 represent overall dissimilarity between pairs, this does not properly describe local similarities because members of the bPBP family are composed of two domains connected by a flexible linker that allows the protein to transition from apo and ligated conformations. SCOP does not discriminate between the two domains; nevertheless, common domain identification algorithms (i.e., PDP [40] and DomainParser [41,42]) do identify the two domains. In Table 2, the PDP domain boundaries are indicated. Note that the large domain is interrupted by insertion of the small one, resulting in two linkers across the domain boundary. The calculated pairwise RMSD between the large and small LAOBP domains individually is 0.4 Å and 0.6 Å, respectively (see Fig. 3b–c). (The corresponding RMSDs between the GBP domains are 0.6 Å and 0.8 Å.) These strong domain-specific similarities indicate that the large and small domains move more or less intact upon ligand binding, which is indicative of a hinge-like motion at the domain boundaries [43].

### Differences within the H-bond networks

Since the mDCM is primarily based on H-bond networks, understanding how they vary is critical to a proper understanding of the model predictions. Initially, we compared H-bond networks by simply counting pairwise topological differences within the networks of two structures. Table 2 describes global H-bond statistics showing the number of H-bonds and average total energy across the four bPBP. The number of H-bonds observed within the four ligated bPBP homologs varies from 327 to 504, which is trivially explained by protein size. The correlation coefficient between H-bond numbers and protein size (number of residues) is 0.99. Surprisingly, in the two examples of ligated and apo structural pairs, the number of H-bonds within the ligated complex is smaller than the number within the apo structure. On average, there are 6.8 H-bonds between bPBP homolog and substrate. Despite the reduction in number of H-bonds on complex formation, the average H-bond strength is significantly increased. In fact, the total H-bond energies for the complex structures are much greater than their corresponding apo structures. These global differences within the H-bond networks will be used below to explain how differences within the model predictions arise.

We found that the above global comparisons failed to provide accurate descriptions of the differences within other QSFR metrics. This failure is likely due to two key issues. First, the employed H-bond energy function [26] is extremely liberal. For example, the energy function frequently identifies very weak H-bonds, such as those between residues *i *and *i+3 *within α-helices (results not shown). They have no pronounced effect on mDCM results as their energies are very small and they rarely contribute anything to the conformational entropy (since they are redundant to other constraints). Nevertheless, the large number of these feeble H-bonds inappropriately skews a distance metric that is based on global statistics. These feeble H-bonds could be filtered out using an energy cut-off, but then we would have to determine this cut-off. Second, due to the nonadditive nature of the mDCM, local topological considerations, which are lost in global metrics, have a considerable effect on output.

**Figure 4:**
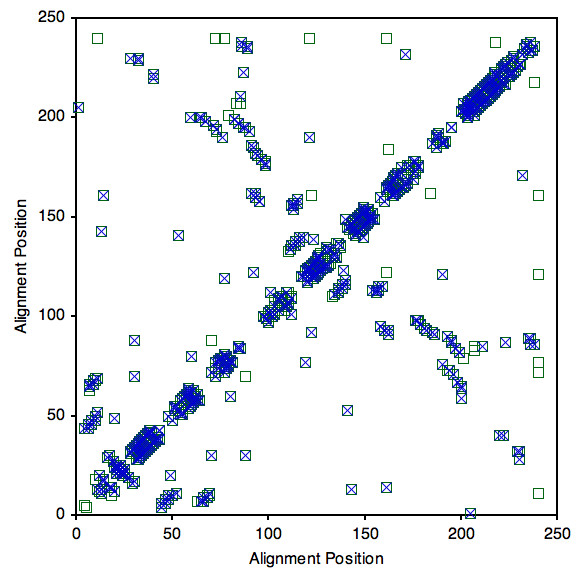
H-bond contact maps of ligated LAOBP (green squares) and HBP (blue x's). Each point on the plot indicates that a H-bond is present between the corresponding residues. Most differences occur between non-secondary structure H-bonds, which primarily involve sidechain interactions and within coil regions.

We opted to employ a simpler but effective approach; where we compare H-bond networks by plotting a H-bond contact map (for example, see Fig. 4) to visualize essential differences. The liberal nature of the H-bond potential, which is primarily manifest within secondary structure elements, can be (visually) ignored because these feeble H-bonds are clustered within a high density of the stabilizing H-bonds. Moreover, the approach straightforwardly highlights where local hydrogen bond topology differences occur. Whether comparisons are made between any two of the four ligated bPBP homologs or between the apo/ligated pairs, key differences in H-bonding mainly occur between non-secondary structure residues. As discussed below, this result has substantial affect on the observed mechanical linkage properties.

### Thermodynamic descriptions

**Figure 5:**
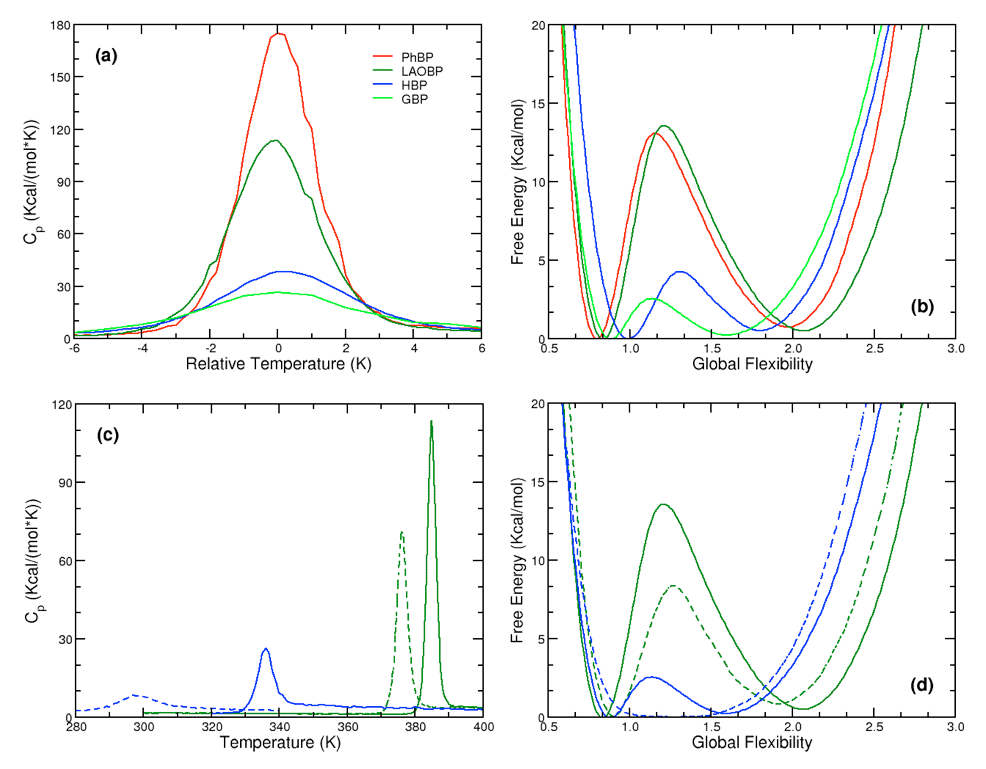
**(a) **Heat capacity curves and **(b) **free energy landscapes of the four ligated bPBP homologs. In **(c) **and **(d) **the heat capacity curves and free energy landscapes, respectively, of the apo structures (dashed lines) are compared to the ligated counterparts. Color coding is conserved throughout.

Employing the HBP best-fit parameters (Table 1) for each of the four ligated structures, their predicted *C*_*p *_curves as a function of temperature, relative to their predicted melting temperature (i.e. *T – T*_*m*_), (see Fig. 5a) shows a high degree of diversity. For example, the maximal *C*_*p *_ranges from 26.4 kcal/(mol·K) for GBP to 174.1 kcal/(mol·K) for PhBP. These differences are not particularly surprising, and, moreover, the underlying H-bond networks explain the variation within the heat capacities. For example, greater H-bond numbers is strongly correlated with *C*_*p*_^*max *^(*R *= 0.90). Similarly, *C*_*p*_^*max *^is also strongly correlated to the number of residues within the protein (*R *= 0.87), which occurs because the number of H-bonds and the number of residues are almost perfectly correlated (*R *= 0.99). Across the four structures, the average number of H-bonds per residue is 1.47 (standard deviation = 0.08). Based on Eq. (4), the effect of H-bond numbers and total H-bond energy on *C*_*p *_can be conceptualized. Greater H-bond numbers provide more opportunities for enthalpic fluctuations to occur, thus increasing the *C*_*p*_. In the same manner, the total H-bond energies, *U*_*hb*_^*max*^, are even more strongly correlated to *C*_*p*_^*max *^(*R *= -0.97). A large part of this relationship is explained by the fact the number of H-bonds is, of course, strongly related to the total H-bond energy (*R *= -0.97). However, the increased correlation to *C*_*p*_^*max *^is due to the greater effect upon the total enthalpy when a stronger H-bond is removed.

The number of H-bonds in each structure is much less strongly correlated to *T*_*m *_(*R *= 0.69); melting points are included in Table 4. The reduced correlation is primarily due to the nontrivial way entropies depend on the distribution of H-bonds, not just simple total numbers. The free energy landscapes, *G*(*θ*) = -*RT*ln*Z*(*θ*), of each of the four structures are plotted at their respective *T*_*m *_in Fig. 5b. Each landscape contains two minima separated by an intervening transition state barrier, indicative of first order (2-state) kinetics. The locations of key free energy landscape features, θ_*nat*_, θ_*TS*_, and θ_*unf*_, are also provided in Table 4. These values are within the range established previously over a structurally diverse set of globular proteins [21]. The heights of the free energy barriers shown in Fig. 5b are strongly correlated to *C*_*p*_^*max *^(*R *= 0.92). The mDCM recapitulates commonly found correlation between barrier height and *C*_*p*_^*max *^when there is two-state kinetics [44]. However, this correlation is not absolutely necessary (neither theoretically nor experimentally). For example, proteins with pronounced *C*_*p *_curves can be barrier free [45], and need not demonstrate cooperative folding/unfolding transitions (e.g., myoglobin [46]).

**Table 4:** Thermodynamic descriptions of the bPBPs.^1^

	LAOBP		HBP	GBP		PhBP
PDB ID	1LST	2LAO*	1HSL	1WDN	1GGG*	1IXH

*T*_*m *_(K)	385	376	336	336	297	386
*C*_*p*_^*max *^(kcal/mol·K)	113.6	70.8	38.5	26.4	8.7	174.1
Barrier (kcal/mol)	13.6	8.4	4.3	2.6	0.0	13.1
θ_nat_	0.8	0.9	1.0	0.8	n/a^2^	0.9
θ_TS_	1.2	1.3	1.3	1.1	n/a	1.2
θ_unf_	2.1	1.9	1.8	1.6	n/a	2.0

^1^All four bPBP homologs have been crystallized in the ligated conformation. Additionally, two bPBP homologs (marked with asterisks) have been crystallized in the open apo conformation. ^2 ^The apo GBP structure is predicted to have a continuous transition between folded and unfolded.

**Figure 6:**
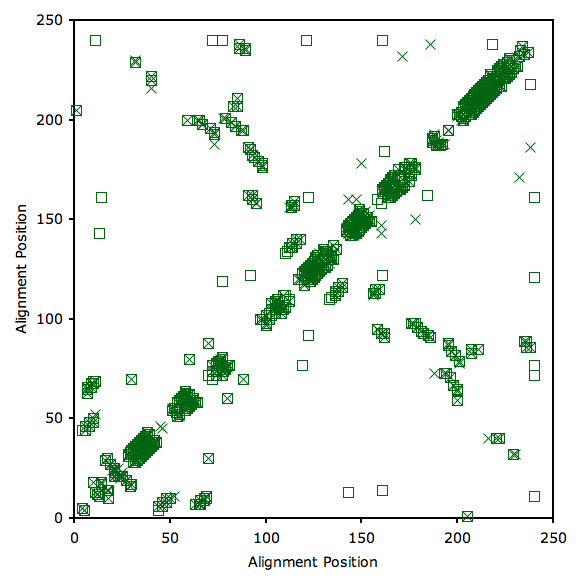
H-bond contact maps of ligated LAOBP (green squares) and apo LAOBP (green x's). Most differences occur between non-secondary structure H-bonds, which primarily involve sidechain interactions and within coil regions.

**Figure 7:**
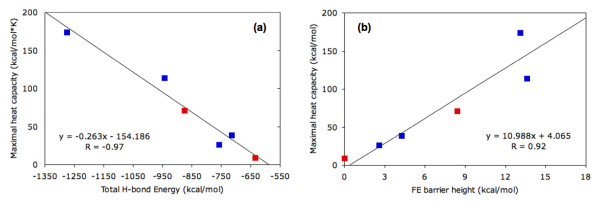
**(a) **Maximal heat capacity vs. total H-bond energy and **(b) **maximal heat capacity vs. free energy barrier height. In both cases, the regression lines, whose equations are provided, and correlation coefficients are computed on the four ligated bPBP homologs (colored blue). The values for the two apo-structures (colored red) are superimposed onto the plot to demonstrate they fit the observed trend.

In addition to the bPBP-ligand complex structures, LAOBP [34] and GBP [47] have been crystallized in the open (apo) forms. Differences within the apo vs. ligated LAOBP hydrogen bond networks are plotted in Fig. 6. Juxtaposed to the variability in *C*_*p *_discussed above, comparison of the ligated and apo structures reveals several clear trends. First, in both cases the *T*_*m *_of the apo structure is reduced compared to its ligated counterpart (Fig. 5c). This predicted down shift for *T*_*m *_when LAOBP and GBP is ligated is fully consistent with experimental observations [27]. The mDCM also predicts *C*_*p*_^*max *^to be substantially lowered upon ligand binding, which arises due to the reduced likelihood of enthalpic fluctuations. This effect is shown in Fig. 7a, where *C*_*p*_^*max *^is plotted versus total H-bond energy for the four bPBP-ligand complexes. The values of the apo structures are superimposed onto the plot of the four complexes, which confirms that the observed changes within *C*_*p*_^*max *^upon ligand removal are strongly associated with the loss of H-bonds. Of course, addition/removal of the ligand is also associated with hydrophobic interactions, free ligand entropy and its chemical potential, all of which are absent within the mDCM. Nevertheless, the importance of the H-bond network as a dominate factor has been pointed out by Cooper [48], showing how the observed *C*_*p *_changes between folded and unfolded protein conformations arise through fluctuations within the H-bond network. Consistent with our results, the same *C*_*p*_^*max *^reduction within the apo form of HBP is observed within the experimental DSC curves [27]. The reduction of *C*_*p*_^*max *^is accompanied by a lower energy barrier (see Fig. 5d). Interestingly, the mDCM predicts a linear relationship between *C*_*p*_^*max *^and free energy barrier height over the six cases studied (Fig. 7b). In fact, the mDCM predicts an unfolding transition with virtually no barrier for apo GBP, suggesting that unfolding/folding is a continuous transition (second order transition). It will be quite interesting to know if future experiments are consistent with this prediction.

### Flexibility along the backbone

**Figure 8:**
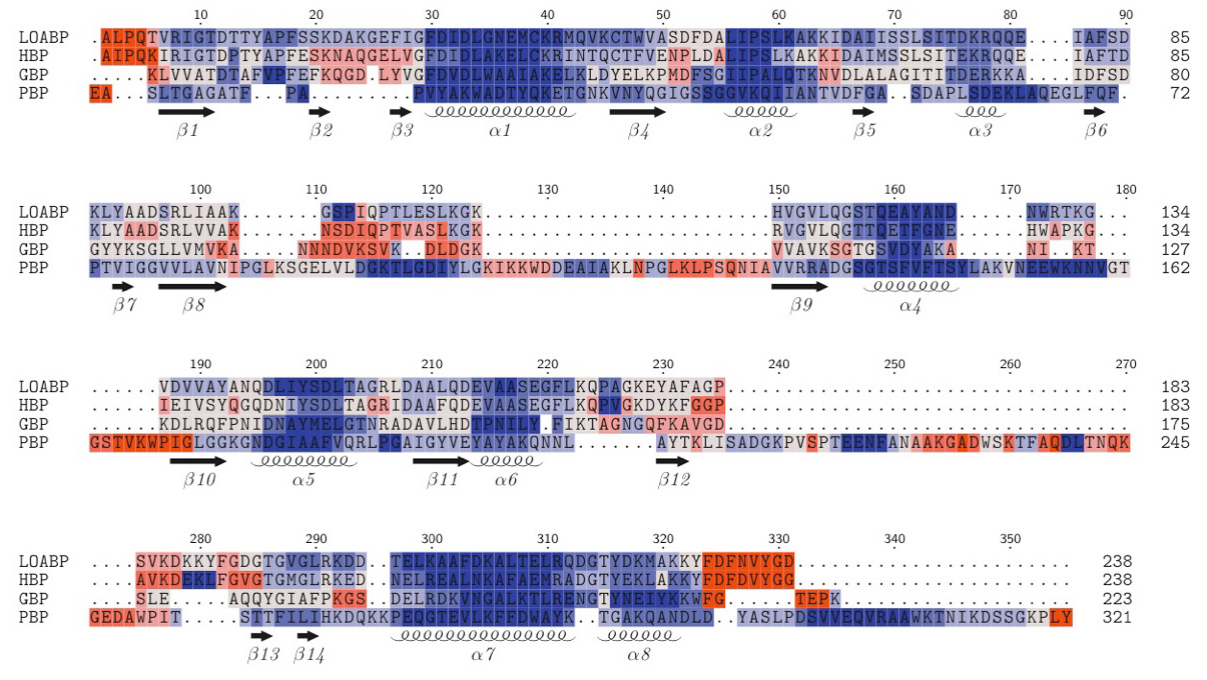
Structural alignment of the four ligated bPBP homologs color-coded by flexibility index. Flexible regions (positive flexibility index) are colored red, whereas rigid regions (negative flexibility index) are colored blue.

**Figure 9:**
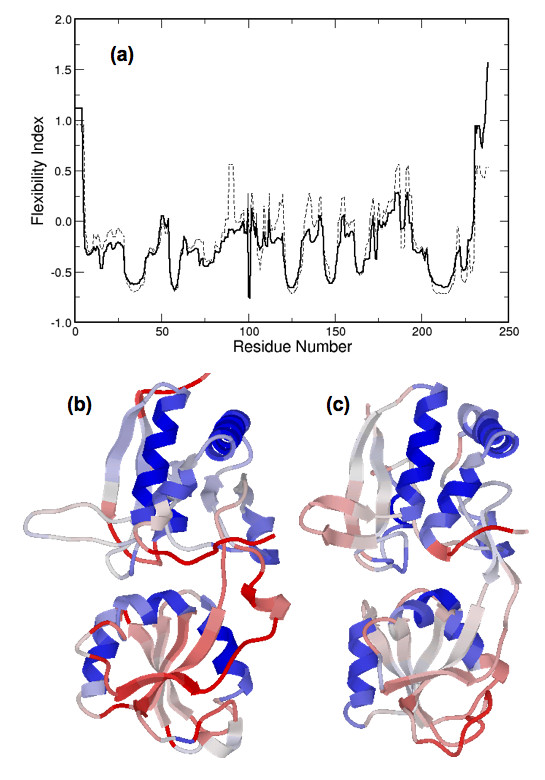
**(a) **Flexibility index of the ligated (blue) and apo (blue) LAOBP. In **(b) **and **(c)**, the flexibility index of the apo and ligated structures, respectively, is mapped to structure. The most pronounced changes occur within the ligand-binding pocket at the hinge between the two domains.

Compared to the large differences within the thermodynamic predictions, predictions of backbone flexibility are more conserved. Fig. 8 color-codes the flexibility index onto the structural alignment of the four bPBP homologs. Regions corresponding to α-helices are predicted to be very rigid, and are the most conserved. Flexibility and rigidity predictions for β-strands are less conserved than found for α-helices. There is large variation in the flexibility index within coil regions, and, as expected, highly flexible regions uniquely occur within the coils. The observed conservation of the backbone flexibility is attributed to the similar H-bond networks within the secondary structure elements. This result is not unexpected as a strong correlation between secondary structure and H/D exchange experiments is well established [49]. It is worth mentioning that our backbone flexibility measures are consistent with those from Elastic Network Models, which are sensitive to fold, but not sequence. Specifically, $\rho_{i}^{idf}$ (the flexible component of the flexibility index) nearly perfectly correlates to the hinge sites predicted by Bahar et al. [50]. These results show that the fold of the protein primarily determines backbone flexibility. Moreover, backbone flexibility is well conserved because of the underlying H-bond sub-network involving secondary structure elements, which are well conserved within a family. As described above, the H-bond network is significantly altered upon complex formation. Consistent with the hinge motion inferred from Fig. 3b–c, differences within the H-bond network between the apo/ligated pair primarily occur within non-secondary structure elements (refer back to Fig. 6). Consequently, backbone flexibility is not significantly affected outside the binding site region, which can be seen in Fig. 9.

### Cooperativity correlation

**Figure 10:**
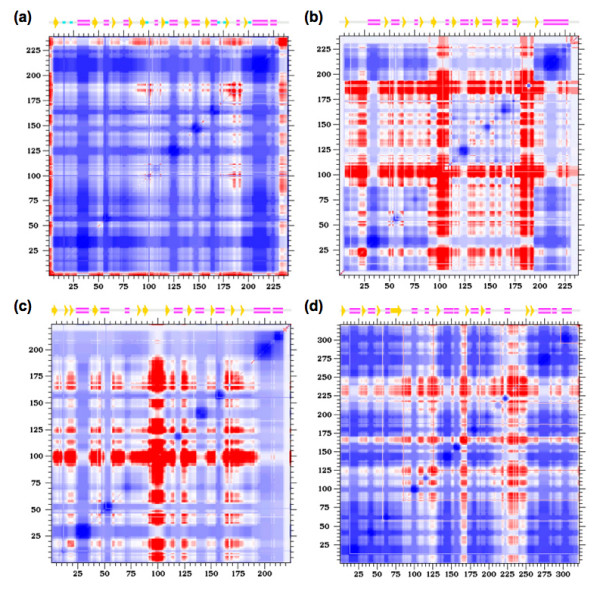
Cooperativity correlation plots of the ligated **(a) **LAOBP, **(b) **HBP, **(c) **GBP, and **(d) **PhBP structures. Red indicates flexibly correlated regions, blue indicates rigidly correlated regions, and white indicates regions of no correlation.

In previous work [22], we demonstrated that cooperativity correlation is moderately conserved across an orthologous RNase H pair. However, this is clearly not the case here; juxtaposed to the flexibility conservation along the backbone, there is immense variation within cooperativity correlation (see Fig. 10). In all cases, the large domain is primarily composed of one large rigid cluster, which results in rigidity correlation in each of the four corners (because the domain is interrupted) of the cooperativity correlation plots. Conversely, the small domain demonstrates much variability within its cooperativity correlation. The small domain generally contains flexibly and rigidly correlated regions within itself and extending into the larger domain. However, the small domain in LAOBP is mostly rigidly correlated with itself and the rest of the protein, indicating that it is primarily composed on one larger rigid cluster. These differences will be discussed again below.

**Figure 11:**
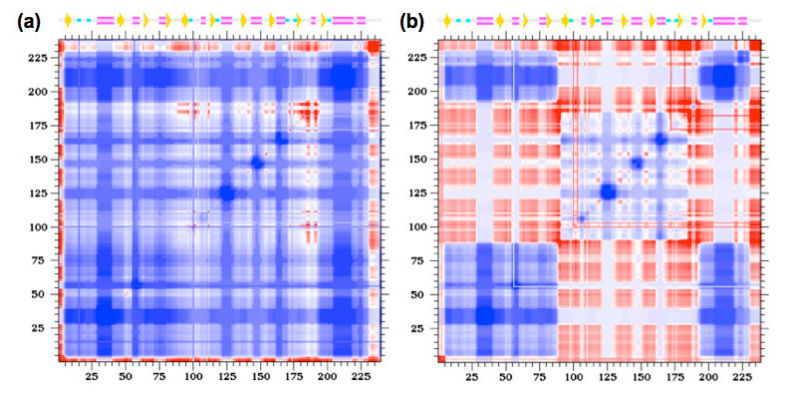
Cooperativity correlation plots of the **(a) **ligated and **(b) **apo LAOBP structures. Note that the color-coding is the same as in Fig. 9, and that panel **(a) **is exactly the same as Fig. 9a.

Differences between the apo/ligated pairs are inline with expectations. In the case of LAOBP, each domain appears as rigidly correlated with itself, whereas the couplings between the two domains are a mix of both *weak *flexible and rigid correlations (Fig. 11). This result is exactly consistent with the inferred hinge motion from the structural analyses discussed above and demonstrated in Fig. 3. In the case of GBP, the apo structure is significantly destabilized (*Δ T*_*m*_*= *39 K). This fact, coupled with the continuous transition observed within apo GBP, results in a cooperativity correlation plot that, as might be expected, is primarily flexibly correlated.

## Discussion

The QSFR results presented demonstrate considerable variability in both mechanical and thermodynamic descriptions of the bPBP family. This observed variability is explained by differences within the underlying H-bond networks. Most notably, variability of thermodynamic quantities correlate well to overall statistical characteristics of H-bond networks, which is consistent with the arguments made by Cooper [48]. Moreover, the predicted reduction of *T*_*m *_upon ligand binding for LAOBP and GBP are qualitatively consistent with the same effect that is experimentally observed for HBP. The observed differences in the thermodynamic response is not particularly unexpected, except, we were initially surprised by the low barrier height found in the free energy landscape for GBP. However, within the context of the mDCM predictions, the total H-bond energy is an excellent indicator for predicting *C*_*p*_^*max*^, and this in return correlates well with barrier height. In the context of this simple extensive H-bond network property (i.e. total energy) over the structures studied, the dramatic deviation from two-state behavior in GBP is not an exceptional case.

The predicted conservation of backbone flexibility is consistent with natural expectations. On the other hand, variability found within cooperativity correlation measures is, at first, somewhat surprising. In prior work we compared mesophilic and thermophilic RNase H orthologs, and the substantial differences in the cooperativity correlation plots (at their respective *T*_*m*_) was contributed to different thermodynamic stability requirements of these two proteins. Nevertheless, after coarse-graining the flexibly correlated regions into contiguous stretches, we identified conserved features presumably needed to support common functional requirements of these two proteins. Our hypothesis that flexibility and rigidity properties of the native state would be conserved across proteins at their corresponding *T*_*m *_within the same family was inconclusive to that study. Starting this work, we maintained the hypothesis that the dominant features in the cooperativity correlations important for function would be markedly conserved. Although general features indicative of the overall fold are indeed conserved, it is the variation that most stands out. Although we can expand the number of proteins to analyze within a family, this report clearly shows that cooperativity correlations are very sensitive to the H-bond network, and the variance in these measures will be the dominate feature. Moreover, other recent results (unpublished) involving nine thioredoxin homologs, also conclude that variability in cooperativity correlation is significant, and sensitive to non-secondary structure elements within the H-bond network.

Clearly, the mDCM, by construction, must have some degree of sensitivity to the characteristics of the H-bond network due to the heavy emphasis it places on H-bonds and the properties of network rigidity. To address the relative importance of H-bond interactions, in ongoing work, we have modified the DCM to deemphasize the affects of native and disordered torsions, and demonstrated that the H-bond interactions within the mDCM account for the bulk of the properties characterizing the thermodynamic transition. It is quite remarkable that despite the simplicity of the mDCM, it captures the essential elements of protein thermodynamics and mechanical linkage mechanisms. However, consider what may happen when more complexity is modeled to explicitly account for solvent effects that include the hydrophobic effect (also work in progress). The likelihood that adding nonspecific interactions will decrease variance in mechanical response is remote. The hydrophobic interactions are of entropic nature related to transfer of water from the interior of a protein to aqueous solution. Based on this simple argument, and having observed no indication to the contrary in alternative model explorations, the H-bond network is expected to remain the key determinant for rigidity and flexibility properties of a protein. Keeping this in mind, we come full circle in understanding why the mDCM flexibility/rigidity predictions are robust despite a greater variance in predicted melting temperatures and other thermodynamic quantities. The nonspecific interactions will be important to modify and perturb protein thermodynamic stability, but the native H-bond network (as determined by X-ray crystallography) will be present in the exact same way as currently modeled in mDCM. We therefore take the view that the mDCM is capturing the most essential element for understanding cooperativity within proteins important for their function.

The H-bond network explains the dichotomy between conservation within backbone flexibility and the lack of conservation within cooperativity correlation. For the most part, pairwise differences within the H-bond networks occur within non-secondary structure (primarily sidechain) H-bonds, meaning that the secondary structure H-bonds are mostly conserved. The conservation within these secondary structure H-bonds is what leads to conserved flexibility along the backbone. Note that backbone flexibility is not exactly conserved; in fact there are many local differences within Fig. 8. These differences presumably arise due to the observed differences elsewhere and the long-range nature of network rigidity [12]. Furthermore, while the secondary structure H-bonds may be conserved within the contact map analysis, this does not mean their energies are equivalent, which also affects the flexibility predictions. Nevertheless, in spite of these nuanced effects, the qualitative conservation within backbone flexibility is a result of the conserved nature of the secondary structure H-bonds.

Compared to local secondary structure H-bonds, differences within sidechain-sidechain H-bonds are expected to result in key differences within the topology of the H-bond networks due to their ability to span across long stretches of sequence. Meaning, a change in just a handful of critically placed non-secondary structure H-bonds can drastically alter mechanical linkage properties. These changes would be most pronounced within the cooperativity correlation plots that explicitly rely on the linkage information. As with the variability within the thermodynamic quantities, the differences between the apo/ligated pairs is used to bolster the argument that the predicted differences are real. In both cases (LAOBP and GBP), the changes upon complex formation are consistent with intuition. Moreover, as we have discussed in previous works [19-22], cooperativity correlation can be interpreted in terms of allostery, and it is a well-known that allosteric response can vary significantly across a family [51-53].

While the arguments above do not prove that trends within the observed predictions are real, they do strongly suggest that this is the case. And, if certain predictions within the current mDCM remain suspect, the above results clearly indicate that future, more sophisticated DCMs should be able to accurately describe trends within mechanical and thermodynamic properties. The results presented here clearly demonstrate how subtle differences within the H-bond networks can lead to unexpected and pronounced complexity. These results are very exciting because it suggests that the paradigm of the DCM is effective in elucidating consequences of altered H-bond networks, for which the computation design of H-bond networks has precedence [54]. More generally, the results presented here suggest that monitoring QSFR will be important to protein design with targeted mechanical and thermodynamic properties, and should be feasible in the near future.

## Abbreviations

ABC – ATP-Binding Cassette; bPBP – bacterial Periplasmic Binding Proteins; ENM – Elastic Network Model; MD – Molecular Dynamics; DCM – Distance Constraint Model; QSFR – Quantitative Stability/Flexibility Relationships; mDCM – minimal Distance Constraint Model; DSC – Differential Scanning Calorimetry; HBP – Histidine Binding Protein; LAOBP – Lysine/Arginine/Ornithine Binding Protein; GBP – Glutamine Binding Protein; PhBP – Phosphate Binding Protein; RMSD – Root Mean Square Deviation

## Authors' contributions

All authors contributed to execution of the presented work. DRL and DJJ planned and oversaw the executed research. DRL, SD, and DJJ wrote the paper. All authors read and approved the final manuscript.

## Supplementary Material

Additional file 1 Supplementary Figure 1. Workflow diagram describing normal usage of the mDCM. Click here for file
