# Supplementary material for: Hydrogen bond networks determine emergent mechanical and thermodynamic properties across a protein family
Source: Chem Cent J. 2008 Aug 12;2:17. doi: 10.1186/1752-153X-2-17 (PMC2533333; doi:10.1186/1752-153X-2-17)
Supplement: Additional file 1 — Supplementary Figure 1. Workflow diagram describing normal usage of the mDCM. [file 1752-153X-2-17-S1.pdf]

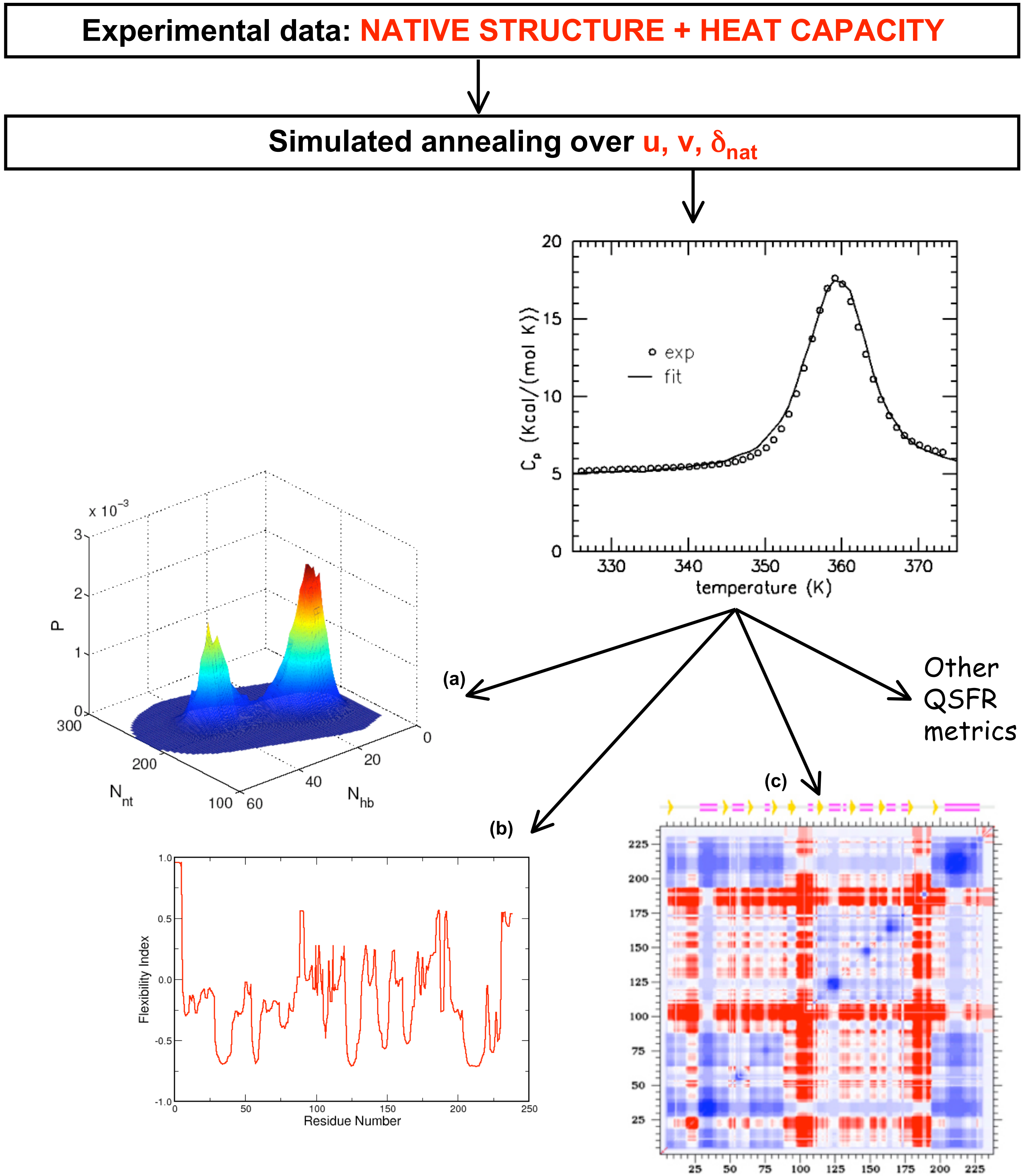


**Supplementary Figure 1:** A generic description of the workflow used by the mDCM. The mDCM begins with two pieces of input: (*i.*) the native structure of a protein and (*ii.*) an experimental heat capacity curve. A simulated annealing process is used to parameterize the model. With mDCM parameters in hand, a large number of mechanical and thermodynamic QSFR metrics can be computed. Three common examples are provided: (a) the free energy landscape (expressed here as probabilities), (b) backbone flexibility, and (c) cooperativity correlation. In the latter two cases, the values represent the thermodynamic average over the full ensemble; however, the mDCM also allows the user to average only specific sub-ensembles (i.e, the native basin only).
